# Supplementary material for: Lactobacillus acidophilus TW01 Mitigates PM2.5-Induced Lung Injury and Improves Gut Health in Mice
Source: Nutrients. 2025 Feb 27;17(5):831. doi: 10.3390/nu17050831 (PMC11901689; doi:10.3390/nu17050831)
Supplement: Supplementary file 1 [file nutrients-17-00831-s001.zip › nutrients-3467838-supplementary.pdf]

Table S1. The histological score of *L. acidophilus* TW01 in DSS colitis mice.

Organ	Histopathological findings	Group				
		**Ctrl**	**NC**	**P**	**TW01 LD**	**TW01 HD**
1.	Lost, crypt, multifocal[1]	0.0±0.0	1.6±1.3*	1.4±0.8*	1.3±0.6*	1.3±0.8*
2.	Regeneration, crypt	0.0±0.0	1.0±0.9*	0.3±0.6	0.5±0.7*	0.9±0.8*
3.	Edema, submucosa	0.0±0.0	1.4±1.4*	0.4±0.9	0.0±0.0#	0.5±0.7*
4.	Inflammation, mononuclear cells	0.0±0.0	1.9±1.5*	1.4±0.8*	1.3±0.6*	1.3±0.8*
5.	Ulcer, with fibroblast cell infiltration	0.0±0.0	1.5±1.4*	0.9±1.1*	0.6±0.5*	0.9±0.8*
	Subtotal mean score[3]	0.0±0.0	1.5±1.3*	0.9±1.0*,#	0.7±0.7*,#	1.0±0.8*,#

Statistically significant difference between the Control and treated groups at *$p < 0.05$. Statistically significant difference between the NC and treated groups at #$p < 0.05$.
